# Unmasking the tumourigenic role of SIN1/MAPKAP1 in the mTOR complex 2

**DOI:** 10.1002/ctm2.1464

**Published:** 2023-10-25

**Authors:** Emilien Ezine, Céleste Lebbe, Nicolas Dumaz

**Affiliations:** ^1^ INSERM U976 Team 1 Human Immunology Pathophysiology & Immunotherapy (HIPI) Paris France; ^2^ Département de Dermatologie Hôpital Saint Louis AP‐HP Paris France; ^3^ Université Paris Cité Institut de Recherche Saint Louis (IRSL) Paris France

**Keywords:** AKT, cancer, MAPKAP1, mTOR, mTORC2, PI3K, signaling pathway, SIN1, targeted therapy

## Abstract

**Background:**

Although the PI3K/AKT/mTOR pathway is one of the most altered pathways in human tumours, therapies targeting this pathway have shown numerous adverse effects due to positive feedback paradoxically activating upstream signaling nodes. The somewhat limited clinical efficacy of these inhibitors calls for the development of novel and more effective approaches for targeting the PI3K pathway for therapeutic benefit in cancer.

**Main body:**

Recent studies have shown the central role of mTOR complex 2 (mTORC2) as a pro‐tumourigenic factor of the PI3K/AKT/mTOR pathway in a number of cancers. SIN1/MAPKAP1 is a major partner of mTORC2, acting as a scaffold and responsible for the substrate specificity of the mTOR catalytic subunit. Its overexpression promotes the proliferation, invasion and metastasis of certain cancers whereas its inhibition decreases tumour growth in vitro and in vivo. It is also involved in epithelial‐mesenchymal transition, stress response and lipogenesis. Moreover, the numerous interactions of SIN1 inside or outside mTORC2 connect it with other signaling pathways, which are often disrupted in human tumours such as Hippo, WNT, Notch and MAPK.

**Conclusion:**

Therefore, SIN1's fundamental characteristics and numerous connexions with oncogenic pathways make it a particularly interesting therapeutic target. This review is an opportunity to highlight the tumourigenic role of SIN1 across many solid cancers and demonstrates the importance of targeting SIN1 with a specific therapy.

## Graphical abstract

- SIN1 is a cornerstone subunit of mTORC2, stabilizing the complex and regulating its substrate specificity.
- SIN1 upregulation and overexpression are associated with many types of cancer and drive tumourigenesis.
- SIN1 is connected with other signaling pathways disrupted in cancer:  Hippo, WNT, Notch, RAS and MAPK.
- SIN1 is an ideal therapeutic target to specifically inhibit mTORC2 in cancer cells

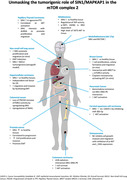

## 1 BACKGROUND

The evolutionarily conserved serine/threonine kinase mTOR (mechanistic target of rapamycin) has emerged over the years as one of the key regulators for integration of upstream signals, such as growth factors, with intracellular signals.^1^ There are many environmental factors that can activate mTOR, from nutrients, oxygen, redox sensors and growth factors, to cellular energy level. As part of the phosphatidylinositol 3‐kinase (PI3K) pathway, mTOR is involved in cellular metabolism, protein translation, ribosome biogenesis and generation of nucleic acids, proteins and lipids. Because mTOR activation is central for cell cycle progression, cell growth, cell proliferation and cell survival, it is often constitutively activated in cancers to sustain tumour growth.^2^, ^3^, ^4^ Activation of the PI3K‐AKT‐mTOR pathway in tumours is due to point mutations or amplifications of kinases (PIK3CA, AKT and MTOR), upstream receptor tyrosine kinases (epidermal growth factor receptor [EGFR], HER2, MET and FGFR) or small GTPases of the RAS family (HRAS, KRAS and NRAS). Moreover, constitutive activation of the PI3K‐AKT‐mTOR pathway can also be due to inactivating mutations or loss of expression of the negative regulators of this pathway (PTEN, PIK3R1, TSC1, TSC2 and LKB1).^2^, ^5^ Due to the frequent alterations of the PI3K‐AKT‐mTOR pathway in a variety of cancers, inhibitors of the kinases PI3K, AKT and mTOR have been developed and tested in clinical trials. Unfortunately, these inhibitors have shown limited efficacy in cancer in part due to the release of potent negative feedback loops causing compensatory overactivation of upstream signaling nodes, including PI3K, AKT and ERK that oppose the antiproliferative effects of the inhibitors. For example, the mTORC1/S6K axis mediates negative feedback of PI3K/AKT activation through inhibition and degradation of the insulin receptor substrate (IRS) docking proteins IRS‐1. Accordingly, suppression of mTORC1 activity by rapalogs prevents inhibitory phosphorylation of IRS‐1 releasing feedback inhibition of PI3K/AKT activation and resulting in disease progression.^6^ Furthermore, mTORC1 directly phosphorylates the adaptor protein growth factor receptor bound protein 10 (GRB10) which is known to suppress signaling induced by insulin and IGFs. As the phosphorylation of GRB10 potentiates its inhibitory activity, acute suppression of GRB10 phosphorylation by rapalogs eliminates its ability to attenuate insulin/IGF signaling thereby leading to MEK/ERK activation.^7^ Therefore, progress in defining the molecular mechanisms underlying mTORC2 function in cancer could pinpoint more selective strategies for targeting the mTOR pathway. Here we highlight the central role of SIN1 as a pro‐tumourigenic factor in mTORC2 and explore the interest of targeting SIN1 in cancers to specifically inhibit mTORC2.

## 2 MTORC1 AND MTORC2

mTOR is the catalytic subunit of two structurally and functionally distinct multi‐protein complexes: mTORC1 and mTORC2. They exert pleiotropic effects under different conditions, primarily through activation of different downstream effectors.

**FIGURE 1:**
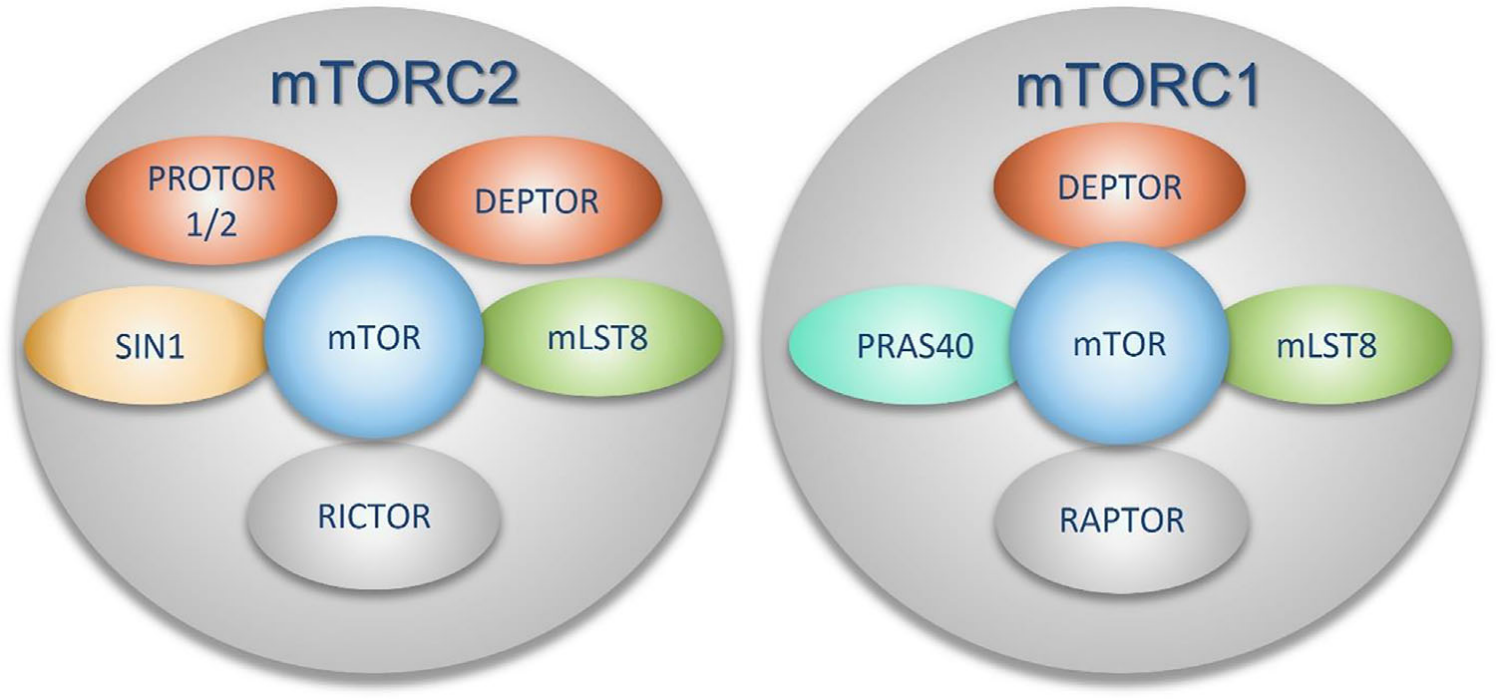
Protein core composition of mTORC1/C2. Scaffold protein in grey, kinase in blue, protein of interest in yellow, green protein role are unclear, orange protein are regulators (activators or inhibitors).

mTORC1 is the most studied complex, formed by the association of 5 proteins: mTOR, the regulatory associated protein of mTOR (RAPTOR), mammalian lethal with Sec‐13 protein 8 (mLST8 also called GβL), proline‐rich AKT substrate 40 Kda (PRAS40) and DEP domain TOR‐binding protein (DEPTOR) (Figure 1).^4^ This complex is sensitive to rapamycin and nutrient status.^8^ When mTORC1 is activated, it stimulates cell growth and cell proliferation by promoting the translation of mRNA into protein by phosphorylating ribosomal protein S6 kinase (S6K) and inhibiting eIF4E binding proteins (4EBP). This complex also regulates the energy metabolism of cells and inhibits autophagy to increase translation, including translation of metabolic enzymes and metabolism‐related transcription factors.^9^ In summary, mTORC1 activates molecular mechanisms that promote cell cycle progression and cell proliferation. Its excessive activation contributes to uncontrolled tumour cell proliferation and tumour growth.

In contrast, mTORC2 is a complex that has historically been less studied, and whose role in tumuorigenesis is less defined. It consists of mTOR, mLST8, DEPTOR, rapamycin‐insensitive companion of mTOR (RICTOR) and SIN1 (also called MAPK associated protein 1 (MAPKAP1), MIP1 or SAPK‐interacting 1). mLST8 plays a role in activation and stabilization of the complex^10^ and the latter is modulated by DEPTOR^11^, ^12^ whereas RICTOR has a scaffolding role^13^ (Figure 1). SIN1 plays a major part in the complex, presenting specific substrates to the mTOR catalytic subunit. It participates in the structure of the complex due to its protein interactions with mLST8, RICTOR and mTOR and allows localization of the complex to membranes via its PH domain (see Section 3 below). mTORC2 responds only partially to rapamycin and is stimulated by growth factors.^14^ When activated, mTORC2 phosphorylates the AGC kinases AKT, SGK and PKC. The AGC kinase family can be activated in general by phosphorylation on three conserved motifs: the turn motif (TM), the hydrophobic motif (HM) (within the c‐terminal tails) and the activation loop (T‐loop) in the kinase catalytic domain.^15^ mTORC2 directly phosphorylates the TM and HM of AGC kinases while PDK1 phosphorylates the T‐loop.^16^, ^17^

**FIGURE 2:**
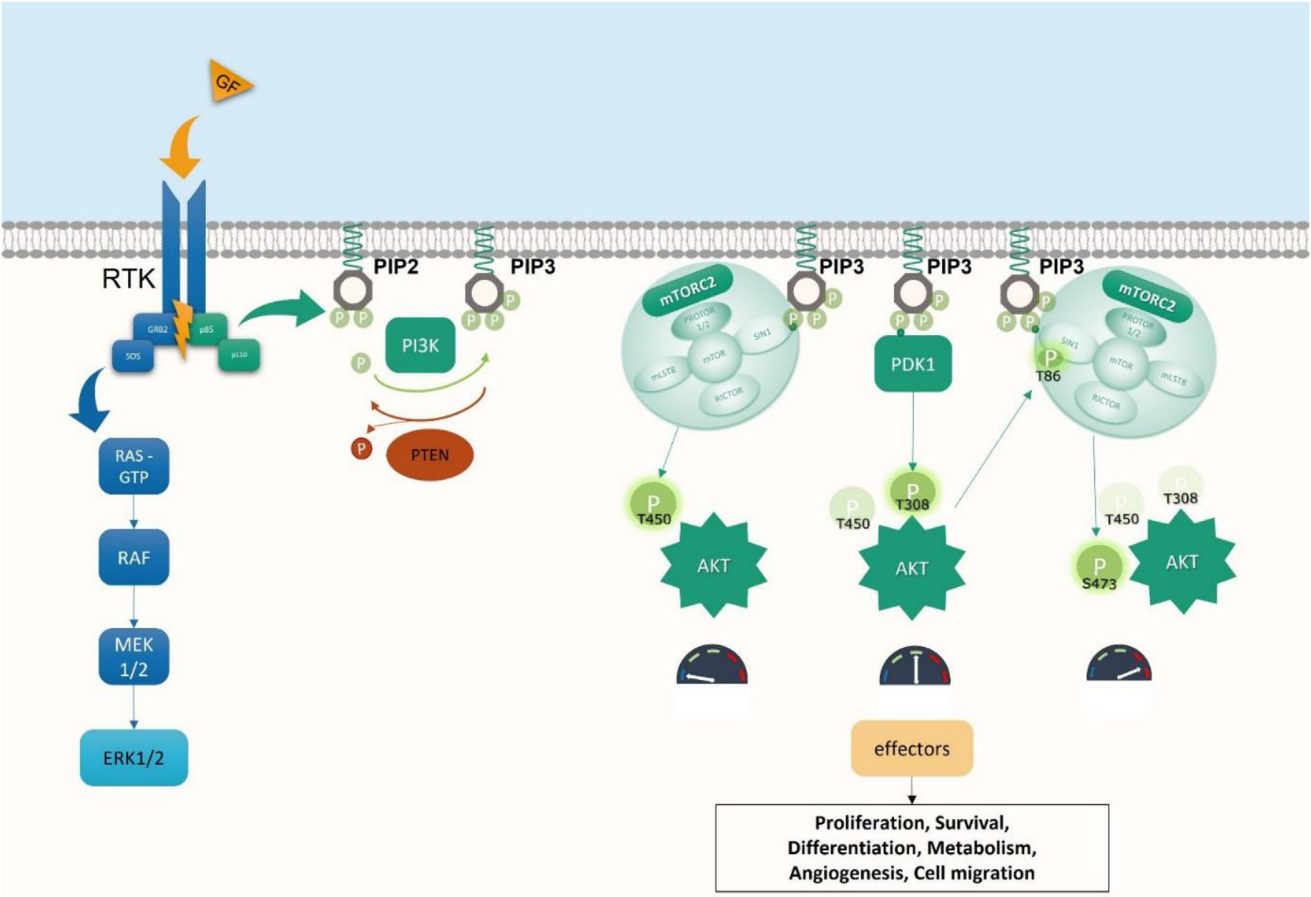
PI3K/AKT/mTOR pathway, MAPK pathway and full AKT activation mechanism. Activation of the PI3K‐AKT‐mTOR pathway (green) is initiated by the activation of various receptor tyrosine kinases (RTK) by their ligands. The catalytic subunits of PI3K are then activated upon binding of its regulatory subunits to the pYXXM motifs on RTKs. Once activated, it phosphorylates the membrane phospholipid PtdIns(4,5)P2 (PIP2) to PtdIns(3,4,5)P3 (PIP3), which functions as a potent second messenger molecule by recruiting to the plasma membrane various kinases such as AKT and PDK1 via their Pleckstrin Homology (PH) domain. Phosphatase and TENsin homolog (PTEN) are a lipid phosphatase that antagonizes the function of PI3K by dephosphorylating PIP3 back to PIP2. Whereas PIP3 directly recruits and activates mTOR complex 2 (mTORC2), AKT is first phosphorylated on his TM (T450) during translation by mTORC2, then on the T‐Loop (T308) by PDK1, increasing its kinase activity. TM and T‐LOOP phosphorylation are responsible of the activation of SIN1 (T86) and finally, mTORC2 is responsible of the last phosphorylation on hydrophobic motif of AKT (S473) for is full activation. MAPK pathway is represented in blue. GF, growth factor.

The best‐characterized kinase of this family is AKT, which is phosphorylated on three sites almost exclusively by mTORC2 and PDK1 (Figure 2). AKT subsequently phosphorylates SIN1 on T86, enhancing mTORC2 kinase activity, which leads to phosphorylation of AKT on S473 (HM) by mTORC2, resulting in full activation.^18^ Once activated, AKT phosphorylates a large number of apoptotic factors, transcription factors or oncogenic factors.

## 3 STRUCTURE AND FUNCTION OF SIN1, AN ESSENTIAL SUBUNIT OF MTORC2

**FIGURE 3:**
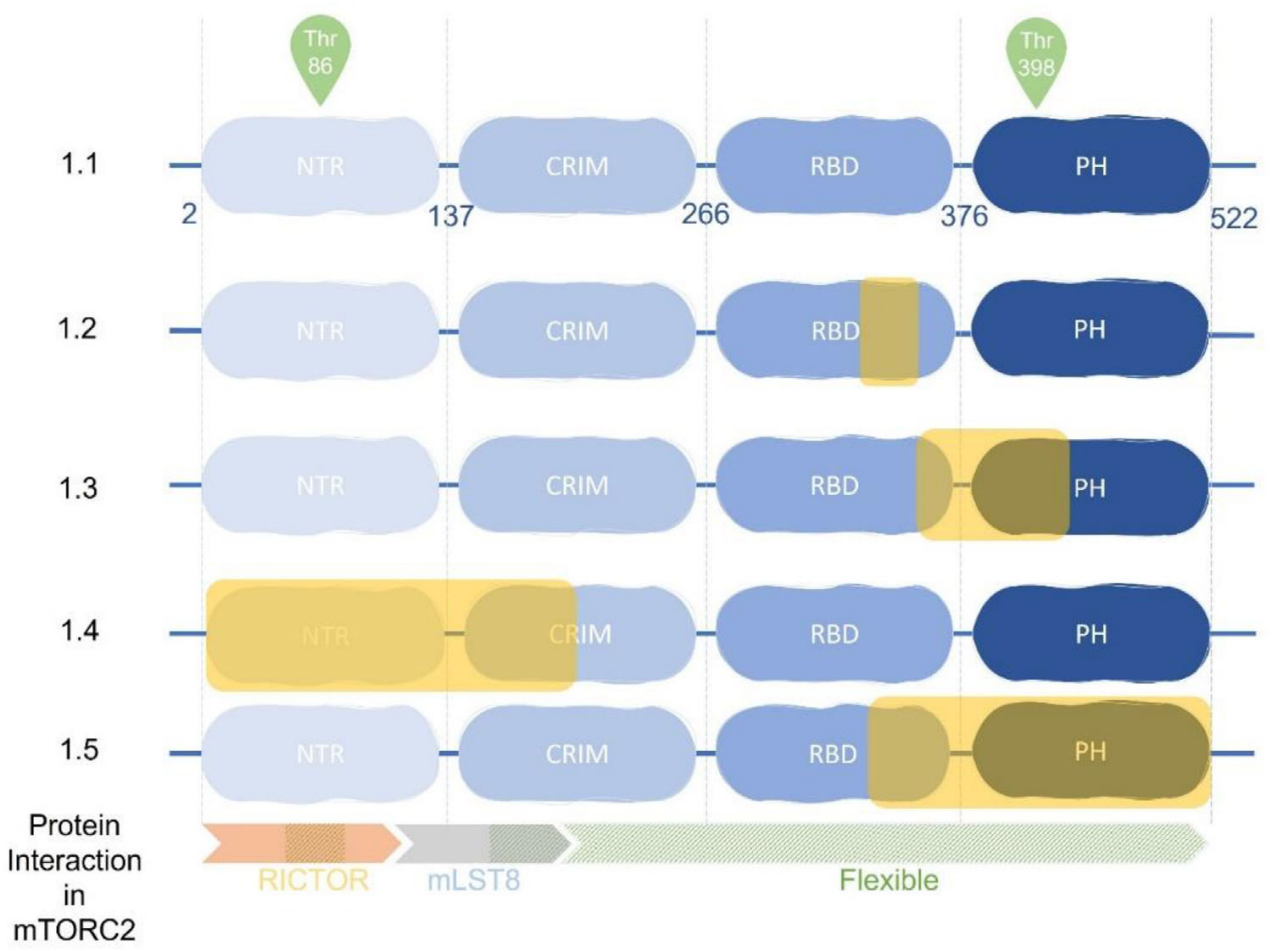
Protein interaction in mTORC2 and specificity of SIN1 isoform. Each domains of SIN1 are represented in blue. Yellow inset represents missing domain parts of isoforms. Orange arrow represents interaction with RICTOR, the gray one with mLST8, and the hatched parts the flexible parts of SIN1.

The *MAPKAP1* gene located on chromosome 9 encodes six splicing variants, due to exon skipping or alternative transcriptional initiations, producing at least five SIN1 isoforms.^19^ The longest isoform presents four distinct regions in its canonical sequence (SIN1.1): the N‐Terminal Region (NTR), the Conserved Region In the Middle (CRIM), the Ras Binding Domain (RBD) and the Pleckstrin Homology domain (PH) (Figure 3 and Table 1).

**TABLE 1:** Transcript variant and isoform of SIN1.

Transcript variant	Synonyms	Isoform	Nt^a^	AA^b^		CCDS^c^
1	SIN1	1.1	3369	522	Canonical isoform	CCDS35140.1
2	SIN1 beta	1.2	1461	486	Lacks an alternate in‐frame exon in the 3′ coding region, compared to variant 1.	CCDS6864.1
3	SIN1 gamma	1.3	1428	475	Lacks an alternate in‐frame exon in the 3′ coding region, compared to variant 1	CCDS35139.1
4	SIN1 delta	1.4	993	330	Has a shorter N‐terminus, compared to isoform 1. The existence of this isoform has not been confirmed experimentally. Variants 4 and 5 encode the same isoform.	CCDS35141.1
5	SIN1 epsilon	1.4	993	330	Also known as SIN1e, lacks two alternate in‐frame exons in the 5′ coding region, and uses a downstream start codon, compared to variant 1. The resulting protein (isoform 4), also known as the delta isoform, has a shorter N‐terminus, compared to isoform 1. The existence of this isoform has not been confirmed experimentally. Variants 4 and 5 encode the same isoform.	CCDS35141.1
6	SIN1 alpha	1.5	972	323	Differs in the 3′ coding region and UTR, compared to variant 1	CCDS48020.1

^a^ number of nucleotides.

^b^ Amino Acid.

^c^ Consensus Coding Sequence.

### 3.1 NTR and CRIM act as scaffold and target recognition

The NTR (residues 2−137 of SIN1.1) contains three distinct sections: a RICTOR‐interacting section, a bridge section connecting RICTOR and mLST8, and an elongated section wrapping around mLST8.^20^, ^21^ This region is essential because it has a scaffolding role and when it is altered, mTORC2 is disrupted.^19^, ^20^, ^21^, ^22^, ^23^, ^24^ The CRIM (residues 138−266) appears to be the signature sequence of the SIN1 family.^25^ While RICTOR plays a central role in blocking access of mTORC1 effectors,^20^ the SIN1‐CRIM domain allows binding of specific substrates to the complex (AGC family) around residues 352−361.^23^, ^26^, ^27^

### 3.2 RAS ‐SIN1 interaction through RBD

The RBD (residues 267−376) is a domain permitting interaction with active members of the RAS family.^28^ RAS‐GTP binding increases the enzymatic activity of mTORC2 in vitro or in cells at the plasma membrane and enables the oncogenic RAS pro‐proliferative cell cycle transcriptional program. Inhibition of SIN1‐RAS interaction decreases the activity of mTORC2 and impairs RAS‐dependent neoplasia in vivo.^29^ Interestingly, recent data show that RAS interaction with SIN1 seems dispensable for mTORC2 activity in physiological conditions. Indeed, human cells and mice expressing a mutant of SIN1 that is unable to bind RAS are proficient for activation and assembly of mTORC2 and for AKT or PKCα phosphorylation.^30^ In conclusion, the interaction between SIN1 and active RAS places SIN1 at the crossroads between the PI3K‐AKT‐mTOR pathway and the MAPK pathway, particularly in cells with oncogenic mutations of RAS which are present in about 30% of human tumours.

### 3.3 Localization and activity of mTORC2 through the PH domain

SIN1 contains a PH domain allowing binding to lipids, which explains the localization of mTORC2 kinase activity to cell membranes: plasma membrane, mitochondria, a subpopulation of endosomal vesicles and on the surface of endoplasmic reticulum.^31^, ^32^ The binding of mTORC2 to PIP3 releases the PH domain of SIN1 and activates mTORC2.^33^ However, Ebner et al. showed that there is a subpopulation of active mTORC2 constitutively present at the plasma membrane, and therefore independent of PI3K activity.^32^ These data suggest that the dynamic partitioning of mTORC2 and AKT could serve to regulate the localization and extent of AKT phosphorylation and signaling in response to growth factors.^32^

### 3.4 SIN1 interactome

**FIGURE 4:**
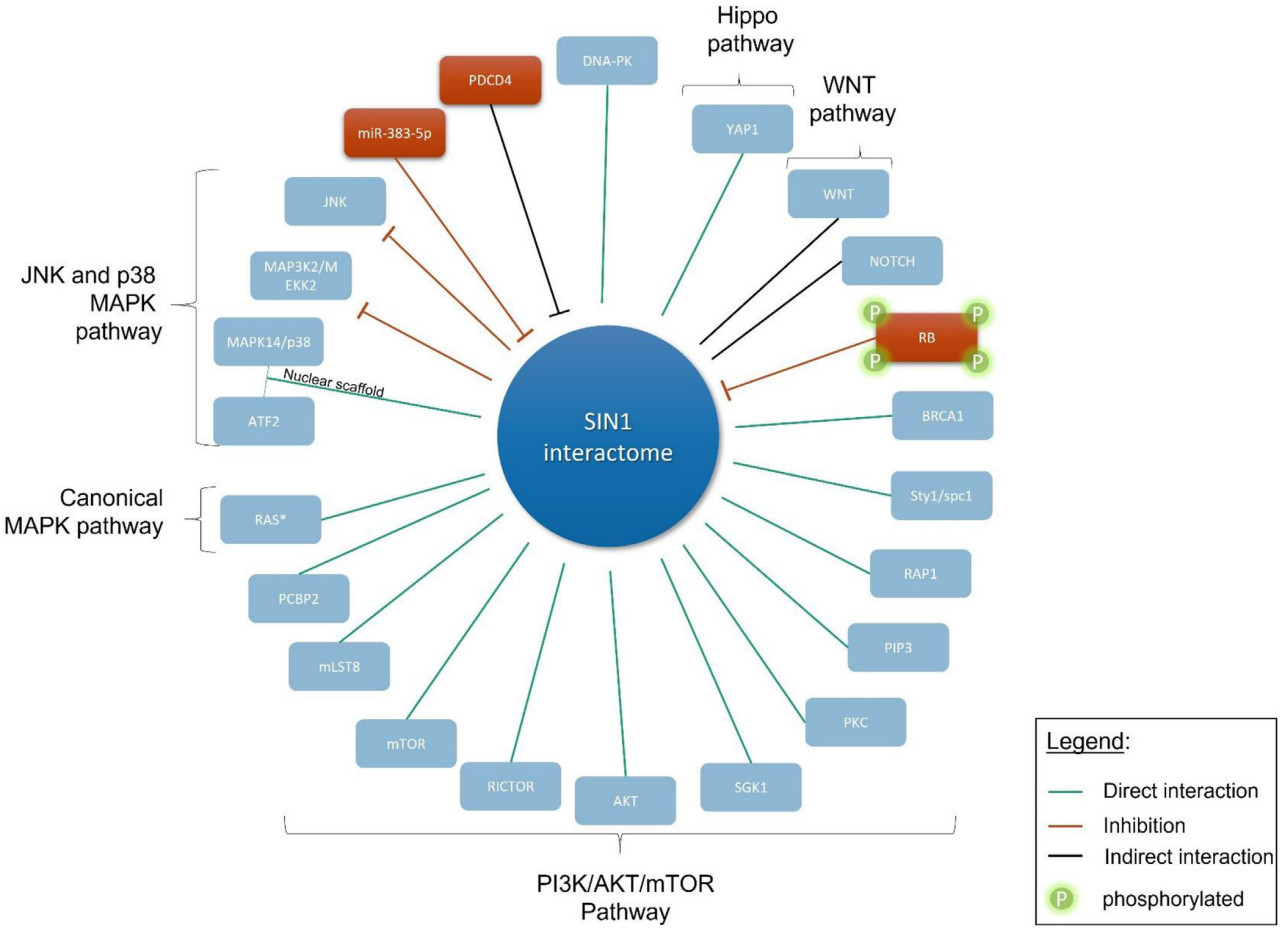
SIN1 interactome. Since the discovery of SIN1 as a subunit of mTORC2, its involvement in the assembly, the specificity of the substrate or the localization of the complex are increasingly studied. These multiple interactions in the complex and outside determine these functions in immunity, metabolism, cell growth. We have represented SIN1 and the various interactions described to date, either as direct (green line) or indirect (black line). When this interaction leads to an inhibition, we represented it in red (rectangle or line). This illustrates the growing interest in targeting mTORC2 via SIN1.

Although SIN1 is better known to be an essential subunit of the mTORC2 complex, acting as a protein scaffolding and modulator of its kinase activity, the partners interacting with SIN1 are very broad (detailed in Table 2 and Figure 4). SIN1 interacts with various stress‐associated kinases^34^, ^35^, ^36^ and small GTPases.^30^, ^37^ Having no enzymatic activity, its role could be summarized as a platform for counteracting the response to stress.^38^

**TABLE 2:** Partners of SIN1 and the interaction effect outside of mTORC2 complex proteins.

Partner	Interaction effect	References
AKT Thr450	Regulates the stability of AKT	^39^
AURKA	Inhibits ubiquitination and subsequent degradation of SIN1	^40^
BRCA1	Interacts with SIN1, RICTOR, PRR5 leading to inhibition of mTORC2 activity	^41^
DNA‐PK	Interact with SIN1 to activate AKT(S473)	^42^, ^43^, ^44^
IFNAR2 /IFNGR1	Interacts with respectively ovin and human SIN1 to activates IFN α,β (IFNAR2) and γ (IFNGR1) transduction pathways	^45^
JNK	Inhibits basal JNK activity and UV‐induced activation	^34^
MAPK14/p38 ATF2	Increases ATF2‐dependent transcription	^36^
MAP3K2, MEKK2	Negatively regulates MEKK2 activation	^35^
PCBP2	Enables selective expression of cell survival factors	^38^
RAP1	Regulates its activation and actin remodeling	^37^
RAS	Activates the proliferative cell cycle transcription program of RAS. Inhibits ERK activation.	^29^, ^46^, ^47^
RB	Interacts with SIN1 to inhibit mTORC2 activity	^48^
Sty1/Spc1	Regulates stress‐dependent transcription	^49^
YAP1	mTORC2 interacts with YAP via sin1, and positively regulates its transcriptional activity	^50^

The first studies on SIN1 in healthy human cells showed an interaction with MEKK2 (MAP3K2). SIN1, which was then called MIP1 for MEKK2 interacting protein1, prevents MEKK2 activation by blocking its dimerisation, which in turn blocks c‐Jun N‐terminal kinase 1 (JNK1), signaling.^35^ SIN1 can also form a complex with JNK in vitro and in vivo and inhibits its activation by UV, suggesting that SIN1 may also act as scaffold molecules in the regulation of signaling by JNK .^34^ Furthermore, SIN1 appears to also function as a scaffolding protein in the SAPK signaling pathway by binding to both p38 and ATF‐2 and enhancing ATF‐2‐dependent transcription.^36^ Finally, an interaction between SIN1 and poly(rC) binding protein 2 (PCBP2) was shown to counteract environmental stress induced by TNFα and H_2_O_2._^38^

Although SIN1 interacts with many proteins and pathways, the role of these interactions in cancer is not fully understood as described below (see Figure 4).

## 4 SIN1 AND CANCERS

**FIGURE 5:**
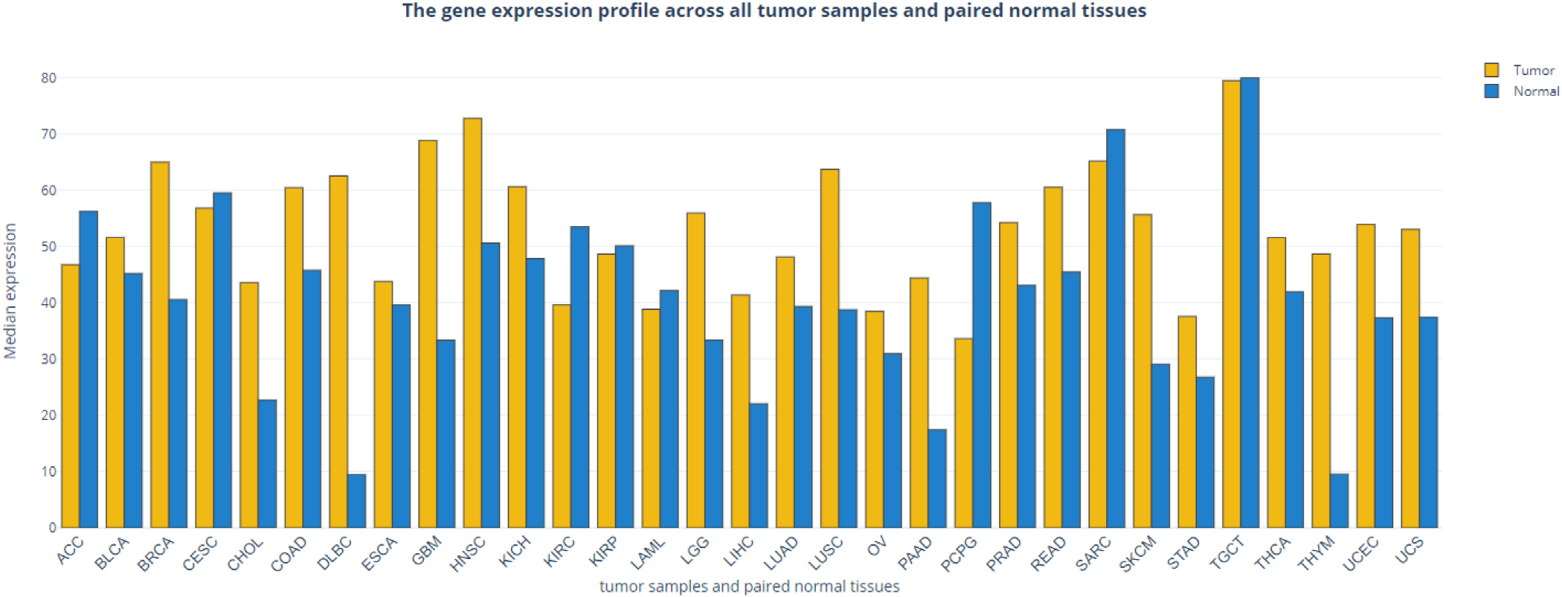
MAPKAP1 expression profile across all tumour samples and paired normal tissues (GEPIA data). Histogram represents median expression of MAPKAP1 in tumour samples and paired normal tissues. ACC, adrenocortical carcinoma; BLCA, bladder urothelial carcinoma; BRCA, breast invasive carcinoma; CESC, cervical squamous cell carcinoma and endocervical adenocarcinoma; CHOL, cholangio carcinoma; COAD, colon adenocarcinoma; DLBC, diffuse large B‐cell lymphoma; ESCA, esophageal carcinoma; GBM, glioblastoma multiforme; HNSC, head and neck squamous cell carcinoma; KICH, kidney chromophobe; KIRC, kidney renal clear cell carcinoma; KIRP, kidney renal papillary cell carcinoma; LAML, acute myeloid leukemia; LGG, brain lower grade glioma; LIHC, liver hepatocellular carcinoma; LUAD, lung adenocarcinoma; LUSC, lung squamous cell carcinoma; MESO, mesothelioma; OV, ovarian serous cystadenocarcinoma; PAAD, pancreatic adenocarcinoma; PCPG, pheochromocytoma and paraganglioma; PRAD, prostate adenocarcinoma; READ, rectum adenocarcinoma; SARC, sarcoma; SKCM, skin cutaneous melanoma, STAD, stomach adenocarcinoma; TGCT, testicular germ cell tumours; THCA, thyroid carcinoma; THYM, thymoma; UCEC, uterine corpus endometrial carcinoma; UCS, uterine carcinosarcoma; UVM, uveal melanoma.

In this section we highlight the literature on SIN1 in human tumours. We searched the PUBMED database (‘SIN1’ OR ‘MAPKAP1’) AND (‘neoplasm’ OR ‘cancer’) and found 179 results. The references of the articles selected were also analyzed. These studies taken separately are not strong enough to draw a conclusion for each cancer. However, taken as a whole, they highlight the importance of this protein in tumourigenesis and raise the question of its therapeutic interest. Overexpression of SIN1 has been found in several cancers (Figure 5) where it was shown to promote proliferation, invasion and metastasis.^51^, ^52^, ^53^, ^54^ In parallel, inhibition of mTORC2 and in particular SIN1 in cancer cell lines was shown to inhibit some of the hallmark of cancers.^55^ Although most data point toward a pro‐tumourigenic role for SIN1, a few publications suggest a tumour suppressor role.^56^, ^57^, ^58^ This discrepancy is not yet elucidated and will require further investigation. The correlation between SIN1 overexpression, tumour progression and poor survival in a variety of cancers is detailed below.

### 4.1 Medullary and papillary thyroid carcinoma

Thyroid carcinogenesis is associated with mutations of BRAF, RAS and RET, and frequent activation of the PI3K‐AKT‐mTOR pathway. Using immunohistochemistry in tissue specimens from patients with thyroid cancers, SIN1 was shown to be overexpressed in all aggressive or poorly differentiated papillary carcinomas. Its expression was much lower in non‐aggressive papillary carcinomas and follicular carcinomas associated with a better prognosis. In thyroid carcinoma cell lines, SIN1 was overexpressed in aggressive papillary thyroid carcinoma (PTC) compared with conventional PTC and cell lines of medullary and anaplastic thyroid carcinoma. SIN1 expression correlated with AKT activation in thyroid carcinomas tissues and cell lines.^59^ SIN1 interacts also with Aurora kinase a (AURKA), an oncoprotein which promotes the proliferation and migration of PTC cells. Mechanistically, AURKA compromises ubiquitination and subsequent degradation of SIN1, leading to hyperactivation of the mTORC2‐AKT pathway in PTC cells.^40^ These findings agree with previous studies showing that AKT is activated in aggressive thyroid tumour types and suggest that SIN1‐dependent AKT activation may be a target for experimental therapy.^60^

### 4.2 Tumours of the central nervous system

Glioma is the most common central nervous system (CNS) malignancy and presents frequent alterations that activate epidermal growth factor receptor (EGFR) and PI3K pathways. In a drosophila model of EGFR and PI3K‐dependent glioma, Read et al. found that orthologous genes of SIN1, RICTOR and CDK4 were key genes for abnormal neoplastic glial proliferation but not for the development of glia.^61^ Holmes et al. described a SIN1‐dependent YAP1 phosphorylation on S436, promoting glioblastoma growth, migratory capacity and invasiveness, both in vitro and in xenograft experiments. mTORC2 was able to regulate YAP activity independently of the Hippo pathway via the interaction of SIN1/YAP.^50^ In a comparison of 27 glioblastoma (GBM) biopsies with six healthy brain samples, a significant correlation was found between the high levels of phospho‐S473‐AKT, S436‐YAP, CTGF and Cyr61. These data agree with a study exploring mTORC2 activity in gliomas and demonstrates that mTORC2 activity was elevated in glioma cell lines as well as in primary tumour cells as compared with normal brain tissue. This increased activity correlated with elevated RICTOR protein and mRNA levels and induced anchorage‐independent growth, increased S‐phase cell cycle distribution, increased motility.^62^ Finally, a proteomic study in pediatric medulloblastoma with a 17p deletion showed that SIN1 and RICTOR were overexpressed three and four times respectively compared to healthy brain tissue, supporting the idea that mTORC2 is a viable therapeutic target in CNS tumours.^63^

### 4.3 Pancreatic cancer

Pancreatic cancer is one of the most aggressive human tumours, with poor prognosis. It is often resistant to standard therapies, including gemcitabine. The DNA‐dependent protein kinase (DNA‐PK) is a key enzyme in this context of DNA‐related toxicity, regulating the resolution of DNA double‐strand breaks via the nonhomologous end joining (NHEJ) pathway.^64^ Gemcitabine resistance could be partly explained by the interaction between SIN1 and the DNA‐PK catalytic subunit (DNA‐PKcs). Inhibition of the SIN1‐DNA‐PKcs complex by DNA‐PKcs knockdown or by inhibitors, prevented AKT phosphorylation and enhanced gemcitabine‐induced cytotoxicity and apoptosis in PANC‐1 cells. Furthermore, SIN1 siRNA‐knockdown also facilitated gemcitabine‐induced apoptosis in PANC‐1 cells. Moreover, in situ, DNA‐PKcs and phospho‐S473 AKT expressions were significantly higher in human pancreatic cancer tissue than surrounding normal tissue.^42^ Together, these results suggest that the interaction between DNA‐PKcs and SIN1 is important for AKT activation and gemcitabine resistance in pancreatic cancer cells.

### 4.4 Breast cancer

Deleterious mutations in the Breast Cancer 1 (BRCA1) gene are associated with an increased risk of breast and ovarian cancer. The tBRCT (tandem BRCA1 C‐terminal) domain of BRCA1 has been shown to interact with SIN1 as well as RICTOR and to reduce mTORC2 activity. The lack of BRCA1 expression could therefore contribute to the hyperactivation of the AKT pathway observed in breast cancer.^41^, ^65^ Krieger et al. showed that mTORC1 and mTORC2 are required for DNA damage‐induced gamma‐H2AX and BRCA foci formation and that mTOR inhibition diminished foci formation. Moreover, mTORC2 activity prevented cisplatin‐induced cell‐death in MCF‐10A cells by activating AKT, and inhibition of mTOR cooperated with cisplatin to induce apoptosis.^41^ In addition, it was shown that the level of SIN1 mRNA expression was significantly upregulated in breast cancer samples compared with normal tissues, and in breast cancer cell lines compared with human breast epithelial cells. Overexpression of SIN1 in MDA‐MB‐468 promoted cell proliferation, colony formation and migration ex vivo and tumour growth in vivo. Conversely, knockdown of SIN1 inhibited proliferation and migration.^51^ Taken together, these results demonstrate that SIN1 plays an important role in breast cancer, in particular for patients lacking functional BRCA1.

### 4.5 Cutaneous carcinoma

Solar ultraviolet (UV) radiation, particularly its ultraviolet‐B (UVB) component, has long been associated with skin carcinogenesis. UVB radiation causes cell DNA damage, along with activation of several signal transduction pathways, including AKT phosphorylation in keratinocytes. Similarly to what was described with gemcitabine in pancreatic cancer, in keratinocytes it was shown that upon UVB radiation, DNA‐PKcs associated with SIN1. This interaction was dependent on EGFR activation and appeared to be required for AKT S473 phosphorylation. siRNA silencing of SIN1, as well as inhibition of EGFR, abolished AKT S473 phosphorylation by UVB. SIN1 interaction with DNA‐PK has been shown to be involved in the resistance of keratinocytes to apoptosis induced by UVB, and their inhibition significantly enhanced UVB‐induced cell death and apoptosis. In conclusion, the data suggest that UVB‐activated DNA‐PKcs forms a complex with SIN1 promoting AKT activation and cell survival, which might be important for tumour cell transformation.

### 4.6 Colorectal cancer

Programmed cell death 4 (PDCD4), is a tumour invasion suppressor frequently downregulated in colorectal cancer. Recent data have shown that the loss of PDCD4 increased the activity of mTORC2 by upregulating SIN1. PDCD4 binds to the translation initiation factor EIF4A to inhibit translation of specific genes. The loss of PDCD4 increases the translation of SIN1 thereby upregulating SNAIL expression and invasion of colorectal cancer cells. Silvestrol, an inhibitor of EIF4A, directly suppressed SIN1 translation and attenuated invasion. Moreover, in colorectal cancer tissues, the SIN1 protein but not mRNA was significantly upregulated, while PDCD4 protein was downregulated, confirming, in colorectal cancer patients, the connection between loss of PDCD4 and increased SIN1 protein level.^54^ Recently, Sane et al. identified a new tumour suppressor in colorectal cancer called UBXN2A, capable of suppressing the mTORC2 signaling pathway via the ubiquitination of RICTOR for 26S proteasomal degradation, without effect on mTORC1.^66^ These data highlight the importance of mTORC2 in colorectal cancer and the therapeutic potential of selective anti‐mTORC2 drugs for these tumours.

### 4.7 Osteosarcoma

Nitidine chloride (NC) is a quaternary ammonium alkaloid that exerts a tumour‐suppressive function in various types of human cancers.^67^ NC inhibits osteosarcoma (OSc) cell growth, invasion and migration, and induces apoptosis partly through inhibition of AKT phosphorylation. Xu et al. showed that NC diminished expression of SIN1 in OSc cells and that overexpression of SIN1 abrogated the inhibition of cell growth and motility induced by NC in OSc. Downregulation of SIN1 by siRNA in combination with NC induced a higher cell growth inhibition compared with NC alone or siRNA alone, suggesting that the mechanism of action of NC may not rely solely on SIN1 inhibition. Thus, NC triggers an anti‐tumour activity in osteosarcoma partially via the inhibition of the SIN1 protein.^68^

### 4.8 Cervical squamous cell carcinoma

Cervical squamous cell carcinoma (CSCC) is the most common cervical cancer. Recent data have shown that CASC9‐1 (Cancer Susceptibility Candidate 9), a newly discovered lncRNA, plays an oncogenic role in CSCC, by upregulating SIN1. SIN1 is the target of miR‐383‐5p which itself is a direct target of CASC9‐1. Therefore CASC9‐1 promotes CSCC cell proliferation, migration and invasion while repressing apoptosis by up‐regulating SIN1 driving AKT phosphorylation. Overexpression of SIN1 totally overturned the effects mediated by CASC9‐1 knockdown confirming that CASC9‐1 pro‐tumourigenic influences on CSCC cells are linked to upregulation of SIN1.^69^ The regulatory mechanism of CASC9‐1/miR‐383‐5p/SIN1 highlights novel therapeutic targets for CSCC treatment.

### 4.9 Prostate cancer

Aberrant Androgen receptor (AR) and PI3K‐AKT signaling are very frequent in prostate cancer (PCa) patients. A recent study showed that SIN1 was highly expressed in tumour tissues compared to normal tissues and that its expression was closely related to PCa progression. SIN1 enhanced PCa cell proliferation and invasion by regulating mTORC2‐AKT pathway and epithelial‐to‐mesenchymal transition (EMT).^70^ Interestingly, the androgen‐mediated mTORC2/AKT activation targeted only a subset of AKT substrates including p27 and FOXO1, but not PRAS40.^71^

### 4.10 Hepatocellular carcinoma

Hepatocellular carcinoma (HCC) has a high rate of metastasis and recurrence, explaining the poor overall survival. Two studies have showed that SIN1 expression levels were 2.2 times higher in HCC tissues than in healthy tissues. High levels of SIN1 were also associated with tumour number, capsular formation and venous invasion, and were an independent risk factor for overall survival (*p* = .046).^52^ SIN1 expression was also upregulated in highly metastatic HCC cell lines, where its inhibition significantly decreased migration and invasion. SIN1 depletion attenuated expression of mesenchymal markers such as SNAIL, VIMENTIN, MMP9 and N‐CADHERIN while it increased the expression of E‐CADHERIN, indicating that SIN1 promotes invasion and metastasis of HCC by facilitating EMT.^52^ Due to its regulation by estrogens, SIN1 expression varies between male and female mice with liver injury and liver cancer, and appears to have distinct roles depending on the risk factors. Considering the risk factors associated with HCC (hepatitis virus infection and alcohol consumption), high SIN1 expression was beneficial for male, but not for female survival.^57^ In conclusion, SIN1 may be a novel biomarker for HCC which is sex‐dependent and sensitive to particular risk factors.

### 4.11 Non‐small cell lung cancer

Non‐small‐cell lung carcinoma (NSCLC) accounts for about 85% of all lung cancers and are largely insensitive to chemotherapy. In NSCLC cells A549 and H1299, overexpression of SIN1 promoted the proliferation and migration of cells while SIN1 knockdown inhibited them. As described in other cancers, overexpression of SIN1 downregulated the expression of epithelial marker (E‐cadherin), while it increased the expression of mesenchymal markers (Vimentin), suggesting that SIN1 promoted migration and invasion of NSCLC cells via induction of EMT. Accordingly, in a xenograft model, SIN1 promoted NSCLC cell tumourigenesis. High expression levels of SIN1 may serve as a novel molecular marker for NSCLC and a promising target for drug development.^53^

### 4.12 SIN1 and resistance to targeted therapies

We highlighted above that SIN1 is involved in resistance to chemotherapy (gemcitabine) in pancreatic cancer through its interaction with DNA‐PKcs. Recent data suggest that SIN1 could also be involved in resistance of tumour cells to targeted therapies. Constitutive activation of AKT is identified in up to 70% of acute myeloid leukemia (AML) patients and mediates, at least in part, the leukemogenic effects of activating fms like tyrosine kinase (FLT3) internal tandem duplication (ITD) mutations. In patients, high SIN1 mRNA levels are significantly associated with poorer event free survival (EFS) and overall survival (OS). Moreover, SIN1 protein levels, assessed by immunohistochemistry (IHC) on bone marrow specimens prior to treatment, correlate with adverse clinical outcomes in a cohort of AML patients treated with equivalent regimens.^72^ In AML cells, SIN1 is frequently overexpressed through transcription regulation by STAT3. Sorafenib, a multikinase inhibitor with activity against FLT3 is used in patients with FLT3 mutations with interesting results but with uncertain evidence of efficacy in terms of survival.^73^ In an ex vivo model of resistance using BA/F3 clones bearing FLT3 mutations conferring resistance to sorafenib, SIN1 was shown to be upregulated and more active. Conversely, inhibition of SIN1 sensitized the resistant BA/F3 clones to sorafenib treatment.^72^ Therefore, SIN1 is involved in AML pathogenesis and may play a role in resistance to targeted therapies.

Neuroblastoma is a heterogeneous disease with a span from spontaneous regression to untreatable progression. The gene encoding the RTK ALK (anaplastic lymphoma kinase) was identified as a neuroblastoma predisposition gene, and constitutive active mutations are found in both germline and somatically acquired neuroblastomas and in ∼20%–43% of relapsed neuroblastoma patients.^74^ Although the RAS‐MAPK pathway is hyperactivated in ALK‐addicted neuroblastoma cells, these cells are resistance to MAPK inhibitors due to a feedback response mediated by SIN1. MAPK inhibition increased SIN1 phosphorylation on T86, enhancing AKT activation and resulting in increased ALK‐addicted neuroblastoma cell survival and growth. Knockdown of SIN1 expression using RNA interference inhibited MAPK inhibitor–induced AKT activation in these cells.^75^ These data highlight a novel mechanism of SIN1 activation by MAPK inhibitors, which may be important in resistance to targeted therapies.

Retinoblastoma (Rb) is a well characterized tumour suppressor, which is frequently deregulated in various cancers. Rb is antagonized by sequential phosphorylation events, initiated by cyclin D‐CDK4/6 complex in early G1 phase. Because CDKs are overactive or CDK‐inhibiting proteins are not functional in many human cancers, CDK inhibitors have been developed to prevent unregulated proliferation of cancer cells. A connection between the CDK/Rb and PI3K pathway was discovered when it was shown that CDK 4/6 inhibitors sensitize PIK3CA mutant breast cancer to PI3K inhibitors.^76^ Mechanistically, Rb interacts with mTORC2 by binding to the PH domain of SIN1 and inhibits the kinase activity of mTORC2. Because only hyper‐phosphorylated Rb interacts with SIN1, inhibition of Rb phosphorylation by CDK inhibitors attenuates Rb suppression on mTORC2 activation, resulting in elevated AKT phosphorylation and activation, conferring resistance to chemotherapeutic drugs. This study therefore provides a rational for the combination of CDK4/6 and mTORC2 inhibitors for better anti‐cancer efficacy in Rb‐proficient cells.^48^

## 5 TARGETING SIN1

mTORC1 inhibitors have shown effects in many neoplasias including Renal Cell Carcinoma, HER2 negative breast cancer and various neuroendocrine tumours.^9^, ^77^ More than forty molecules inhibiting the signalling pathway PI3K/AKT/mTOR have been studied and are currently at different stages of clinical development: mTOR allosteric or kinase inhibitors, pan‐PI3K inhibitors, dual pan‐PI3K plus mTOR inhibitors, isoform‐specific PI3K (α, β, γ or δ inhibitors) or AKT inhibitors. But only four have been approved for clinical use: temsirolimus and everolimus (mTORC1 inhibitors), idelalisib (PI3K δ inhibitor) and copanlisib (pan‐PI3K inhibitor).^77^, ^78^ As monotherapies, they have shown limited efficacy in certain diseases due to feedback loops and significant toxicities limiting their use to subtherapeutic doses.^6^, ^79^

To date, there is no specific mTORC2 inhibitor available in the clinic; however several authors have shown the benefit of targeting it specifically.^26^

Nevertheless, the importance of the mTORC2 in metabolism,^80^, ^81^ proliferation^51^, ^53^, ^70^ cytoskeleton^82^, ^83^ and immune response^84^, ^85^ suggest that inhibiting SIN1 could impact the normal cellular functioning of non‐tumour cells. However, by specifically targeting protein‐protein interactions within mTORC2, it may be possible to selectively inhibit the oncogenic functions of this complex while reducing off‐target effects, improving the safety profile compared to mTOR inhibitors.

Several studies demonstrated the interest of targeting specific domains of SIN1. Cameron et al., generated a truncated mutant of SIN1 with an intact NTR, but without the CRIM/RBD/PH domain. Although this mutant incorporates into the endogenous mTORC2, it disrupts substrate recruitment to mTORC2. They showed that this mutant inhibited AKT phosphorylation in vivo and in vitro, and decreased tumour size in a colon cancer model, with no impact on mTORC1 activity.^26^

Specifically inhibiting the interaction of SIN1 with RAS could be interesting because whereas it was recently shown that the SIN1‐RBD is not essential for the physiological function of mTORC2 in healthy cells,^30^ several authors have demonstrated that disrupting the RAS‐mTORC2 interaction impaired RAS‐dependent tumour growth in vivo.^29^ In tumoural cells, oncogenic RAS binds to the SIN1‐RBD and this interaction not only promotes the kinase activity of the mTORC2 at the plasma membrane but also activates the pro‐proliferative cell cycle transcription program of RAS.^86^, ^87^, ^88^ Moreover, the crystal structure of the RBD‐SIN1 and RAS interaction has been published allowing the characterization of the amino acids important for this interaction and opening the way to peptide inhibition.^29^, ^30^, ^46^, ^47^, ^89^

Cell‐penetrating peptides (CPPs) are short peptides (less than 30 residues) capable of actively or passively crossing cell membranes. They can transport chemical compounds, large proteins and even nucleic acids.^90^ Thus, a peptide based on the RAS interaction zone with SIN1 and competing with the RBD domain of SIN1 could be interesting for selectively target *RAS*‐mutated tumours. In addition, inhibition of SIN1 could sensitize cells to DNA damaging agents due to the complexation of SIN1 with DNA‐PKC in the context of DNA damage response to ionizing radiation, topoisomerase inhibitors or UVB radiation.^43^, ^44^, ^84^ Inhibition of DNA‐PKC/SIN1 was shown to attenuate DNA damage–induced AKT activation and increase cell death.^43^, ^44^ Inhibition of SIN1 could therefore be combined with radiotherapy to promote cell death and reduce resistance in radio‐resistant cancers.

Several studies have shown that SIN1 plays an essential role in immune function and in the biological response to interferon (IFN). Targeted disruption of SIN1 leads to decreased activation of STAT1 signaling pathway and type I IFN‐induced gene transcription in antiproliferative responses.^91^ SIN1 has also been shown to regulate IFNγ‐induced gene expression and type II IFN‐mediated biological responses via AKT activation and STAT1 tyrosine phosphorylation.^92^ Given the importance of the IFN/STAT1 pathway in the induction of PD‐L1 and therefore the response to anti‐PD1, it will be interesting to study the effect of SIN1 inhibition on the response to immunotherapies.

## 6 CONCLUSIONS

Cancer is a daunting complex multifactorial disease, involving acquisition of capacities that can be summarized in the holistic tools proposed by Hanahan as the “hallmarks of cancer.”^55^ Dysregulation of PI3K‐AKT‐mTOR is central to several of these hallmarks such as enabling senescent cells, evading growth suppressors, sustaining proliferative signaling, deregulating cellular metabolism and resisting cell death. Consequently, mTORC2 as a major actor of the PI3K pathway, plays a central role in tumourigenesis. This review summarizes the present understanding of mTORC2 signaling and functions, focusing on the tumourigenic functions of SIN1 and highlighting the current status and future perspectives for targeting mTORC2 in cancer treatment. This review provides a rationale for developing inhibitors targeting SIN1 to specifically inhibit mTORC2. We previously reviewed the role of RICTOR, the scaffold of mTORC2, in tumourigenesis and resistance to targeted therapies.^13^, ^93^ This review focused on SIN1 because it is a cornerstone subunit of mTORC2, allowing the assembly of mTORC2, stabilizing the complex and regulating its substrate specificity.

SIN1 upregulation and overexpression are associated with many types of cancer,^51^, ^59^, ^61^, ^72^ where it drives hyperproliferation and metastasis.^50^, ^51^, ^52^, ^53^, ^59^, ^70^ The expression level of SIN1 is associated with invasive and aggressive tumours accompanying strong AKT activation.^26^, ^50^, ^59^ Moreover SIN1 promotes epithelial mesenchymal transition,^53^, ^70^ and is responsible for resistance to chemotherapy or targeted therapy in several cancers.^42^, ^72^ SIN1 is also connected with other signaling pathways often disrupted in human tumours such as Hippo,^50^ WNT,^94^ Notch^95^ and MAPK (Figure 4). It therefore seems to be the ideal therapeutic target for numerous types of cancer, which could benefit from specific mTORC2 inhibitors. Because mTOR catalytic inhibitors do not discriminate between mTORC1 and mTORC2, the challenge is to identify therapeutic strategies to selectively block mTORC2 leaving the activities of mTORC1 intact to avoid the inhibition of the feedback loops caused by rapalogs. Designing a specific chemical inhibitor for SIN1 could be a challenge since it has no direct enzymatic activity. However, it may be possible to use a degradation approach by targeted proteolysis such as PROTACs (PROteolysis TArgeting Chimeras).^96^ Another possible approach is to target specific protein‐protein interactions in the mTORC2 complex as was recently demonstrated for small molecules inhibiting the association of mTOR with RICTOR.^97^ These molecules inhibited the phosphorylation of mTORC2 targets AKT, NDRG1 and PKCα without affecting the phosphorylation of the mTORC1 substrate p70S6 kinase.^97^ We suggest that targeting the interactions of SIN1 with its partners could effectively inhibit AKT and cross‐talk between the PI3K pathway and other oncogenic pathways, while avoiding the off‐target effects of mTOR inhibitors. Interestingly, targeting SIN1 may also have positive side effects outside of cancer, as it was recently shown that inhibiting SIN1 had a cardioprotective role in a model of induced hypoxia.^98^

## CONFLICT OF INTEREST STATEMENT

The authors declare no relevant financial or non‐financial interests to disclose.

## Data Availability

All data generated or analyzed during this study are included in this published article.
